# Preparation of Benzothiazole-Substituted Carbosilane Dendrimers up to the 7^th^ Generation

**DOI:** 10.3390/molecules14041605

**Published:** 2009-04-21

**Authors:** Chungkyun Kim, Hyojeong Kim

**Affiliations:** Department of Chemistry, Dong-A University, Busan 604-714, Korea; E mail: hjkim@upchem.co.kr (H.K.)

**Keywords:** Dendrimer, Carbosilane, PL, GPC, Silicone

## Abstract

Carbosilane dendrimers with 2-(2-phenyloxy)benzothiazole groups on the periphery were prepared from the 1^st^ to the 7^th^ generation. All dendrimers were characterized by ^1^H- and ^13^C-NMR, elemental analysis, MALDI TOF MS, GPC, and PL (photoluminescence) spectroscopy. Characteristic PDI (Polydisperse Index) values of the peaks corresponding to the respective dendrimers in the GPC data is in very narrow range of 1.00~1.04. All PL spectra show a blue-shift increasing with generation from the 1^st^ to the 7^th^.

## 1. Introduction

Confirmation of dendrimer isomolecularity has been a controversial subject due to their architectural similarity, especially in the case of higher generations [1,2,3]. At this point the preparation and identification of defect-free dendrimer with uniform size is very important. The isomolecularity of low generation dendrimers, with molecular weights around a few thousand Daltons, has been confirmed by mass spectroscopy [4,5,6]. However this still does not provide any decisive information about structural defects of higher generations. Herein, novel carbosilane dendrimers (1^st^ to 7^th^ generation), having benzothiazoyl group chromophores on the periphery were synthesized and characterized. Uniformities in size of these carbosilane dendrimers were determined by GPC (Gel Permeation Chromatography) [7,9] and their dendritic effects were studied by PL (photoluminescence) spectroscopy.

## 2. Results and Discussion

**Scheme 1:**
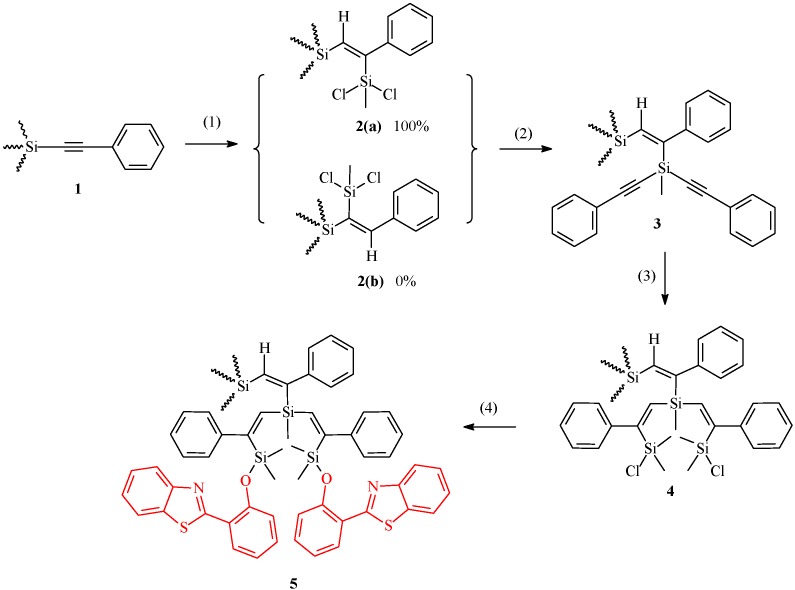
Schematic view of the preparative methods. (1) Hydrosilation between dichloromethylsilane and dendritic branches, (2) Alkynylation, (3) Hydrosilation between chlorodimethylsilane for termination, and (4) Addition of benzothiazole.

Hydrosilation of bis-(phenylacetylenyl)dimethylsilane with hydrosilane and substitution of chlorine in the Si-Cl bond by phenylacetylide are well described in previous papers [5,6]. The same hydrosilation and substitution reaction were repeated for the preparation of dendrimers from generations 1 to 7. They are quite stable in regular atmosphere and are readily soluble in organic solvents. Purification of reaction products by silica gel-toluene chromatography gives the respective dendrimers in high purity and they were characterized by ^1^H- and ^13^C-NMR, elemental analysis, MALDI TOF MS, GPC, and PL (photoluminescence) spectroscopy [8]. The 7^th^ generation, having 256 phenylacetylenyl groups on the periphery was isolated and confirmed as having no structural defects; however, there is no evidence for the formation of the 8^th^ generation. It seems to be that there is enough space for 256 phenylacetylenyl groups on the periphery of the 7^th^ generation, but not enough space for 512 phenylacetylenyl groups on the periphery of the 8^th^ generation [10]. Since the 2-benzothiazolephenyloxy group is bulkier than a phenylethynyl group, only one chlorine atom of the terminal silyl group can be substituted by a 2-benzothiazoylphenyloxy group from the 1^st^ to 7^th^ generation of nG[2,2^n-1^,1]-2^n^BT (n=1~7). These were prepared by the substitution of Si-Cl bonds on the dendritic periphery with 2-(2-hydroxyphenyl)benzoxazole in the presence of a base such as triethylamine and TMEDA, etc. (Scheme 1). 

**Scheme 2:**
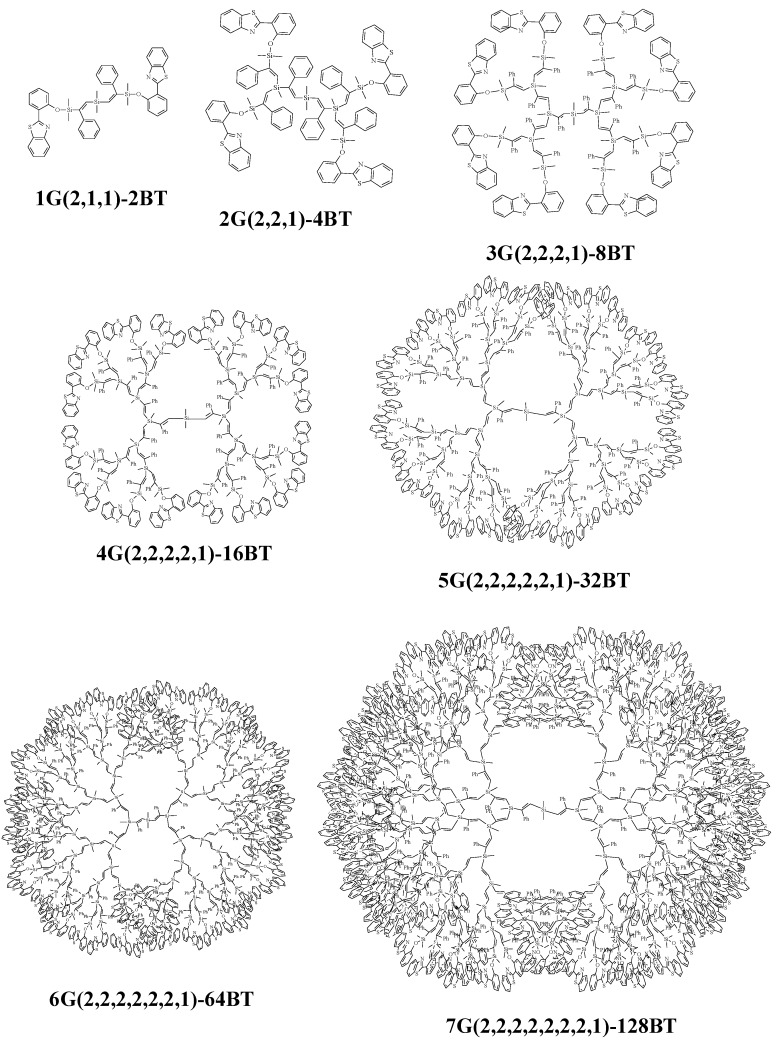
Schematic view of the benzothiazole 1st to 7th generation dendrimers .

**Figure 1:**
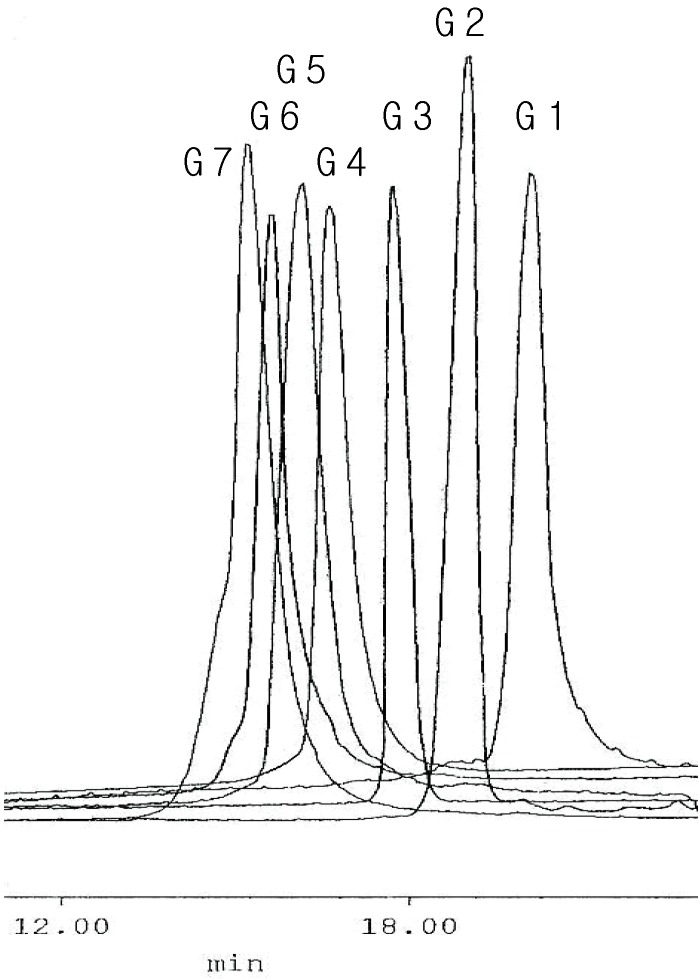
GPC chromatogram of 1^st^ to 7^th^ generation carbosilane dendrimers.

The molecularity of the respective dendrimer of nG[2, 2^n-1^,1]-2^n^BT (Scheme 2) is determined by GPC, in which very narrow peak of PDI value closely to 1.00 is observed at shorter retention time with increasing the generation number (Figure 1). 

MALDI mass spectroscopy provides valuable information about the isomolecularity of lower generation dendrimers (1^st^ generation, 1G[2,0,1]-2BT); however it gives no information about its purity and molecularities for higher generations. Therefore GPC has been used for the characterization of dendrimers along with various spectroscopic measurements and elemental analysis. The GPC chromatogram in Figure 1 shows very narrow peaks corresponding to the respective generations of the dendrimer. The first peak from the left is that of the highest generation dendrimer, 7G[2,2,2,2,2,2,2,1]-126BT with a molecular weight 67,736 Daltons, and the last peak from the left is that of the lowest generation dendrimer, 1G[2,0,1]-2BT (Mw: 831.27). The most significant feature of the chromatogram is that each peak has no shoulder in the left and the right side of the peak for the 1^st^ to the 5^th^ generation dendrimers. On the other hand for the 6^th^ and 7^th^ generation, a little shoulder can be recognized only on the left side of each peak. The shoulder in the left side of the peak must come from the corresponding dendrimer with impurities, which are stuck between its branches. It is plausible that a higher generation dendrimer has more probability to capture any impurities between its branches. Nevertheless no shoulder in the right side of each peak is observed. In addition the PDI values of the peaks are very close to 1.00 in the range from 1.00 to 1.04. It means that the calculated molecular weight from GPC is very slightly greater than that of the dendrimer (*M_w_*≥*M_n_*) [9].

**Scheme 3:**
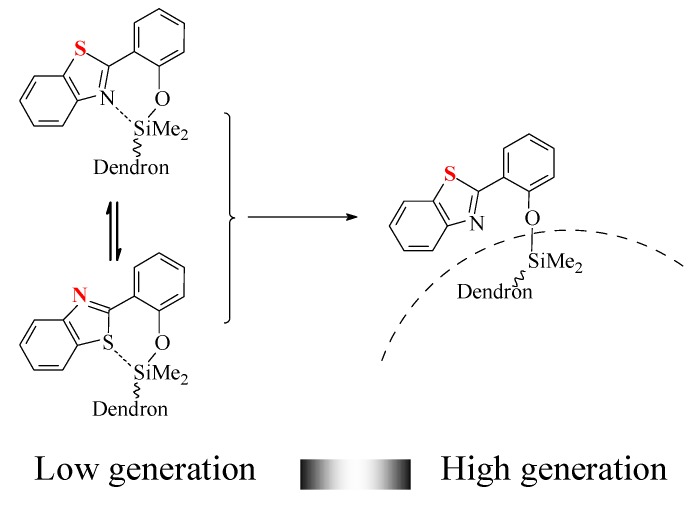
Position of benzothiazole on dendritic periphery.

The PL spectra of nG[2,2^n-1^,1]-2^n^BT ( n = 1 ~ 6) show one smaller λ_max_ peak at the wavelength of 380 nm and the other bigger one at the wavelength of 512 nm. As the generation of nG[2,2^n-1^,1]-2^n^BT increased from the 1^st^ to the 4^th^ generation the intensity of the smaller λ_max_ at the wavelength of 380 nm unexpectedly increased slowly, but the intensity of the bigger λ_max_ at the wavelength of 512 nm is decreased. In the end the intensity of λ_max_ at the wavelength of 380 nm is bigger than that of λ_max_ at the wavelength of 512 nm for the 5^th^ and the 6^th^ generation of nG[2,2^n-1^,1]-2^n^BT (n=5, 6) dendrimers. This can be attributed to the fact that the space around the silicon atoms becomes smaller as the generations increase. In the lower generation dendrimers there is enough space around the silicon atoms for the bulky 2-benzothiazolephenoxy group to occupy a planar geometry, in which the nitrogen or sulfur atoms can coordinate equatorially with a silicon atom. However, in the higher generation dendrimers 2-benzothiazole group is too bulky to move around the Si atoms, therefore the benzothiazole and phenyl groups rotate to minimize the steric hindrance (Scheme 3). The same pattern of the PL spectra are observed for the dendrimers of nG[2,2^n-1^,1]-2^n^BO ( n = 1 ~6) [10]. 

**Figure 2:**
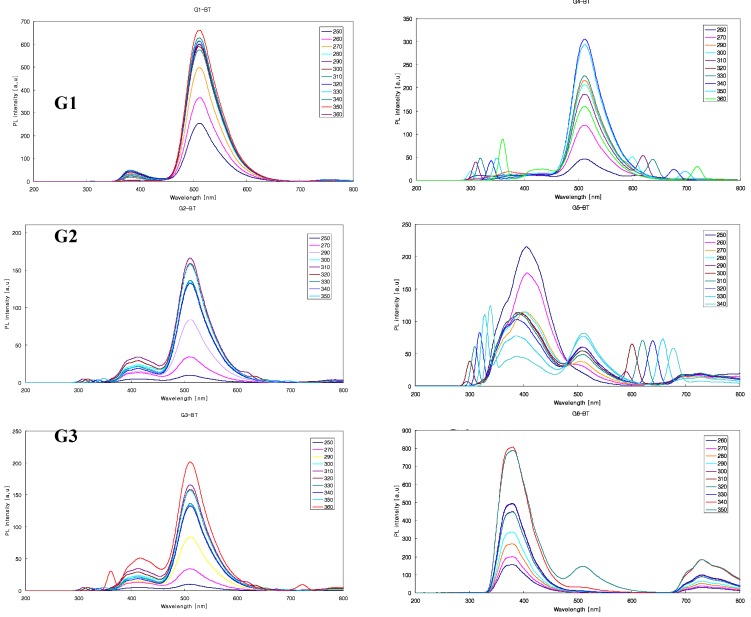
PL properties of benzothiazole dendrimers from 1^st^ to 6^th^ generations.

## 3. Experimental

All reactions were carried out under a dried nitrogen atmosphere attached to vacuum line. NMR spectra were recorded on a Bruker AC 200 instrument. UV spectra were measured with a HP 8452A diode array ultraviolet visible spectrophotometer. The gel permeation chromatography (GPC) was performed in THF at 25 ^o^C with a Waters 515 HPLC pump and a Waters 2410 Refractive Index Detector connected with three columns (Ultrastyragel 0.78 x 30 cm; 10^3^, 10^4^ and 10^5^), which were calibrated with a narrow molecular weight polystyrene standard. Elemental analyses were carried out at KBSI in Daegu, Korea. The following abbreviations are used: BT refers to benzothiazole, PA refers to phenylethynyl groups and TMEDA refers to tetramethylethylenediamine. In the representation of nG[2,2^n-1^,1]-2^n^X, the nG (specially Gn in figure and experimental part) refers to generation number, the first 2 in bracket refers to the number of branch in core, the 2^n-1^refers to the number of branches in inner shell and last 1 in the bracket refers to the number of functional groups on the periphery and last 2^n^BT refers to the number of benzothiazole groups on the whole periphery Gn-mCl refer to number of Si-Cl (m=2,4,..) bonds in n^th^ generation. The hydrosilation and alkynylation processes were described in previous reports [5,10]. 

*1G*[2,1,1]-*2BT*: 1G[2,0,1]-2Cl (0.30 g, 0.67 mmol) in toluene (25 mL) was slowly added to a mixed solution of 2-(2-hydroxyphenyl)benzothiazole (0.33 g, 1.45 mmol) and TMEDA (0.17 g, 1.45 mmol) in dried toluene (50 mL) at room temperature. Then, the reaction mixture was warmed and kept at 60 ^o^C for 1.5 hr. The reaction mixture was filtered to remove the TMEDA-HCl salt. 1G[2,0,1]-2BO was purified and isolated by silica gel chromatography with chloroform-hexane (3:2) as eluent. Yield: 0.44 g (0.53 mmol, 80%) of a light yellow glass. ^1^H-NMR (ppm, CDCl_3_): δ = -0.42 (s, 6H, SiMe, G0), 0.42 (s, 12H, SiMe, G1), 6.42 (s, 2H, CH=C, G0), 6.84~7.00, 7.04~7.22, 7.22~7.39, 7.39~7.54, 7.80~7.90, 8.05~8.15, 8.42~8.54 (26H, BT, Ph); ^13^C-NMR (ppm, CDCl_3_): δ = -0.56 (SiMe, G1), -0.08 (SiMe, G0), 116.97, 118.04, 119.71, 121.68, 122.35, 125.72, 126.15, 126.86, 127.53, 127.63, 127.98, 128.04, 128.58, 132.77, 132.93, 144.69, 152.02, 158.09, 164.34, 169.55 (BT, Ph); Anal. Calc. for C_48_H_46_N_2_O_2_S_2_Si_3_ (Mw: 831.28): C, 69.29%, H, 5.54%, N, 3.37%, S, 7.71%. Found: C, 68.77%, H, 5.56%, N, 3.40%, S, 7.71%; MALDI TOF MS, Calc.: 831.28, Found: 831.27 (M^+^); UV/Vis, λ*_max_* = 287 nm, ε*_max_* = 0.34×10^5^; GPC: PDI value (Mw/Mn), 1.01 (682/674); Rt, 19.96 mins. 

*2G*[2,2,1]-*4BT:* The same method as described for the preparation of G1-2BT was used with G2-4Cl (0.45 g, 0.40 mmol), TMEDA (0.19 g, 1.71 mmol) and 2-(2-hydroxyphenyl)benzoxazole (0.39 g, 1.71 mmol). Yield: 0.59 g (0.31 mmol, 78%) of a light yellow glass.^1^H-NMR (ppm, CDCl_3_): δ = -0.66 (s, 6H, SiMe, G0), -0.54 (s, 6H, SiMe, G1), 0.34 (s, 24H, SiMe, G2), 6.02 (s, 2H, CH=C, G0), 6.32 (s, 4H, CH=C, G1), 6.70~6.89, 7.02~7.15, 7.15~7.40, 7.40~7.55, 7.80~7.89, 8.02~8.12, 8.40~8.54 (64H, BT, Ph); ^13^C-NMR (ppm, CDCl_3_): δ = -2.47 (SiMe, G0), -0.53 (SiMe, G1), 1.23 (SiMe, G1), 116.97, 118.04, 119.71, 121.68, 122.35, 125.75, 126.17, 126.90, 127.64, 127.69, 127.96, 127.99, 128.62, 132.77, 132.93, 144.56, 144.48, 152.09, 158.09, 164.25, 169.59 (BT, Ph); Anal. Calc. for C_112_H_104_N_4_O_4_S_4_Si_7_ (Mw: 1,894): C, 71.03%, H, 5.49%, N, 2.96, S, 7.71%. Found: C, 70.98%, H, 5.54%, N, 3.04%, S, 6.77%; UV/Vis, λ*_max_* = 287 nm, ε*_max_* = 0.70×10^5^; GPC: PDI value (Mw/Mn), 1.01 (910/895); Rt, 19.14 mins. 

*3G*[2,2,2,1]-*8BT:* The same method as that of G1-2BT was used with G3-8Cl (0.49 g, 0.20 mmol), TMEDA (0.20 g, 1.75 mmol) and 2-(2-hydroxyphenyl)benzothiazole (0.40 g, 1.75 mmol). Yield: 0.68 g (0.17 mmol, 85%) of a light yellow glass. ^1^H-NMR (ppm, CDCl_3_): δ = -0.82 (s, 6H, SiMe, G0), -0.75 (s, 12H, SiMe, G2), -0.51 (s, 12H, SiMe, G1), 0.30 (s, 48H, SiMe, G3), 6.00~6.12 (s, 6H, CH=C, G0~G1), 6.22 (s, 8H, CH=C, G2), 6.64~6.86, 6.96~7.12, 7.12~7.39, 7.39~7.54, 7.76~7.89, 8.04~8.15, 8.40~8.52 (134H, BT, Ph); ^13^C-NMR (ppm, CDCl_3_): δ = -2.92 (SiMe, G0~G1), -0.61 (SiMe, G3), 0.87 (SiMe, G2), 116.97, 118.04, 119.71, 121.68, 122.35, 125.75, 126.17, 126.90, 127.64, 127.69, 127.96, 127.99, 128.62, 132.77, 132.93, 144.56, 144.48, 152.09, 158.09, 164.25, 169.59 (BT, Ph); Anal. Calc. for C_240_H_220_N_8_O_8_S_8_Si_15_ (Mw: 4,016): C, 71.71%, H, 5.47%, N, 2.78%, S, 6.37%. Found: C, 71.00%, H, 5.44%, N, 2.30%, S, 6.38%; UV/Vis, λ*_max_* = 287 nm, ε*_max_* = 1.43×10^5^; GPC: PDI (Mw/Mn), 1.01 (3,241/3,197); Rt, 17.87 mins. 

*4G*[2,2,2,2,1]-*16BT:* The same method as that of G1-2BO was used with G2-4Cl (0.32 g, 0.061 mmol), TMEDA (0.12 g, 1.06 mmol) and 2-(2-hydroxyphenyl)benzothiazole (0.24 g, 1.71 mmol). Yield: 0.42 g (0.051 mmol, 83%) of a light yellow glass. ^1^H-NMR (ppm, CDCl_3_): δ = -0.86 (s, 18H, SiMe, G0, G2), -0.75 (s, 24H, SiMe, G3), -0.57 (s, 6H, SiMe, G1), 0.26 (s, 96H, SiMe, G4), 6.04~6.19 (s, 14H, CH=C, G0~G2), 6.21 (s, 16H, CH=C, G3), 6.56~6.85, 6.85~7.14, 7.14~7.36, 7.36~7.50, 7.72~7.84, 8.00~8.12, 8.40~8.50 (278H, BT, Ph); ^13^C-NMR (ppm, CDCl_3_): δ = -2.87 (SiMe, G0~G2), -0.85 (SiMe, G4), 0.89 (SiMe, G3), 116.97, 118.04, 119.71, 121.68, 122.35, 125.75, 126.17, 126.90, 127.64, 127.69, 127.96, 127.99, 128.62, 132.77, 132.93, 144.56, 144.48, 152.09, 158.09, 164.25, 169.59 (Ph, BT); Anal. Calc. for C_496_H_452_N_16_O_16_S_16_Si_31_ (Mw: 8,264): C, 72.02%, H, 5.47%, N, 2.71%, S, 6.19%. Found: C, 71.83%, H, 5.59%, N, 2.21%, S, 6.18%; UV/Vis, λ*_max_* = 287 nm, ε*_max_* = 2.74×10^5^; GPC: PDI (Mw/Mn), 1.04 (4,311/4,114); Rt, 16.69 mins. 

*5G*[2,2,2,2,2,1]-*32BT:* The same method as that of G1-2BO was used with G5-32Cl (0.40 g, 0.037 mmol), TMEDA (0.14 g, 1.20 mmol) and 2-(2-hydroxyphenyl)benzothiazole (0.27 g, 1.20 mmol). Yield: 0.51 g (0.030 mmol, 82%) of a light yellow glass. ^1^H-NMR (ppm, CDCl_3_): δ = -0.95~-0.48 (96H, SiMe, G0~G4), 0.23 (192H, SiMe, G5), 5.84~6.10, 6.10~6.32 (62H, CH=C, G0~G4), 6.55~6.82, 6.82~7.16, 7.16~7.34, 7.34~7.52, 7.68~7.84, 7.96~8.27, 8.38~8.55 (566H, BT, Ph); ^13^C-NMR (ppm, CDCl_3_): δ = -2.86 (SiMe, G0~G4), -0.84 (SiMe, G5), 117.03, 118.10, 119.73, 121.72, 122.41, 125.75, 126.10, 126.90, 127.61, 127.94, 128.62, 132.82, 132.96, 144.69, 152.02, 158.09, 164.34, 169.55 (Ph, BT); Anal. Calc. for C1008H916N32O32S32Si63 (Mw: 16,760): C, 72.17%, H, 5.46%, N, 2.67%, S, 6.11%. Found: C, 71.82%, H, 5.60%, N, 2.67%, S, 6.11%; UV/Vis, λ*_max_* = 287 nm, ε*_max_* = 5.37×10^5^; GPC: PDI (Mw/Mn), 1.02 (8,222/8,201); Rt, 16.09 mins. 

*6G*[2,2,2,2,2,2,1]-*64BT:* The same method as that of G1-2BO was used with G2-4Cl (0.32 g, 0.015 mmol), TMEDA (0.12 g, 1.06 mmol) and 2-(2-hydroxyphenyl)benzothiazole (0.24 g, 1.06 mmol). Yield: 0.43 g (0.013 mmol, 83%) of a light yellow glass. ^1^H-NMR (ppm, CDCl_3_): δ = -1.76~-0.66 (s, 192H, SiMe, G0~G5), 0.08~0.38 (s, 384H, SiMe, G6), 5.72~6.28 (126H, CH=C, G0~G5), 6.48~6.80, 6.80~7.12, 7.12~7.32, 7.32~7.48, 7.62~7.80, 7.97~8.10, 8.30~8.52 (1142H, BT, Ph); ^13^C-NMR (ppm, CDCl_3_): δ = -1.23 (SiMe, G0~G5), -0.43 (SiMe, G6), 117.03, 118.10, 119.74, 121.73, 122.41, 125.76, 126.91, 127.92, 128.34, 128.63, 132.83, 132.97, 144.69, 152.02, 158.09, 164.34, 169.55 (Ph, BT); Anal. Calc. for C_2032_H_1844_N_64_O_64_S_64_Si_127_ (Mw: 33,752): C, 72.24%, H, 5.46%, N, 2.65%, S, 6.07%. Found: C, 71.54%, H, 5.89%, N, 2.64%, S, 6.20%; UV/Vis, λ*_max_* = 287 nm, ε*_max_* = 1.08×10^6^; GPC: PDI (Mw/Mn), 1.01 (11,536/11,397); Rt, 15.55 mins. 

*7G*[2,2,2,2,2,2,2,1]-*128BT:* The same method as that of G1-2BO was used with G7-128Cl (0.27 g, 0.0062 mmol), TMEDA (0.10 g, 0.88 mmol) and 2-(2-hydroxyphenyl)benzothiazole (0.20 g, 0.88 mmol). Yield: 0.36 g (0.0053 mmol, 86%) of a light yellow glass. ^1^H-NMR (ppm, CDCl_3_): δ = -1.02~-0.60 (s, 384H, SiMe, G0), 0.18~0.40 (s, 768H, SiMe, G7), 5.72~6.28 (254H, CH=C, G0~G6), 6.40~7.46, 7.46~7.82, 7.82~8.10, 8.29~8.54 (2294H, BT, Ph); ^13^C-NMR (ppm, CDCl_3_): δ = -1.27 (SiMe, G7), - 0.53 (SiMe, G0~G6), 117.84, 121.16, 125.29, 122.78, 124.56, 125.29, 125.89, 126.69, 127.77, 128.22, 129.03, 129.76, 131.22, 131.80, 135.78, 142.86, 144.127, 152.04, 153.21, 163.13 (Ph, BT); Anal. Calc. for C_4080_H_3700_N_128_O_128_S_128_Si_255_ (Mw: 67,736): C, 72.28%, H, 5.46%, N, 2.64%, S, 6.05%. Found: C, 71.87%, H, 5.90%, N, 2.62%, S, 6.02%; UV/Vis, λ*_max_* = 287 nm, ε*_max_* = 2.27×10^6^; GPC: PDI (Mw/Mn), 1.02 (15,206/14,890); Rt, 15.20 mins.

## References

[1] Tomalia D.A., Hedstrand D.M., Ferritto M.S. (1991). Comb-burst dendrimer topology: new macromolecular architecture derived from dendritic grafting. Macromolecules.

[2] Vögtle F., Gestermann S., Hesse R., Schwierz H., Windischb B. (2000). Functional dendrimers. Prog. Polym. Sci..

[3] Inoue K. (2000). Functional dendrimers, hyperbranched and star polymers. Prog. Polym. Sci..

[4] Higuchi M., Shiki S., Ariga K., Yamamoto K. (2001). First Synthesis of Phenylazomethine Dendrimer Ligands and Structural Studies. J. Am. Chem. Soc..

[5] Kim C., Seo W., Oh M.-J. (2007). Synthesis of Silafluorene on Dendritic Periphery. Bull. Korean Chem. Soc..

[6] Kim C., Kim H. (2005). Synthesis of Farnesyl Terminated Carbosilane Dendrimer. Synthesis.

[7] Trollsås M., Hedrick J.L. (1998). Dendrimer-like Star Polymers. J. Am. Chem. Soc..

[8] Feng X.-H., Taton D., Ibarboure E., Chaikof E.L., Gnanou Y. (2008). Janus-Type Dendrimer-like Poly(ethylene oxide)s. J. Am. Chem. Soc..

[9] Li S., McGrath D.V. (2000). Effect of Macromolecular Isomerism on the Photomodulation of Dendrimer Properties. J. Am. Chem. Soc..

[10] Kim C., Kim H., Oh M.-J., Hong J.-H. (2009). Preparation and unequivocal identification of chromophores-substituted carbosilane dendrimers up to 7th generations. Bull. Kor. Chem. Soc..

